# Ultra‐Processed Food Intake and its Association With Eating Disorders: A Scoping Review

**DOI:** 10.1002/erv.70144

**Published:** 2026-07-04

**Authors:** Marthina Streda Walker, Natália R. Figueiredo, Luana Cristina Lermen, Maria Laura da Costa Louzada, Ana Maria P. Feoli

**Affiliations:** ^1^ School of Health and Life Sciences Postgraduate Program in Psychology Pontifical Catholic University of Rio Grande do Sul (PUCRS) Porto Alegre Brazil; ^2^ Department of Nutrition School of Public Health University of São Paulo São Paulo Brazil; ^3^ Center for Epidemiological Research in Nutrition and Health University of São Paulo São Paulo Brazil

**Keywords:** anorexia nervosa, binge eating disorder, bulimia, eating disorders, industrialised foods, ultra‐processed foods

## Abstract

**Objective:**

Ultra‐processed foods have been associated with adverse health outcomes, but their relationship with eating disorders remains unclear. This scoping review mapped the available evidence on the association between ultra‐processed food intake and eating disorders in adolescents and adults.

**Method:**

Following Joanna Briggs Institute recommendations and PRISMA‐ScR guidelines, searches were conducted in PubMed, PsycINFO, and Embase up to March 2026. Eligible studies were original quantitative research assessing ultra‐processed food intake, defined by the Nova classification or equivalent descriptions, in relation to eating disorders. Two reviewers independently conducted study selection, data extraction, and methodological appraisal.

**Results:**

Of 930 records identified, seven cross‐sectional studies met the inclusion criteria, comprising 47,758 adolescents and adults. Across studies, positive associations were more often reported for nonrestrictive symptoms, including binge eating, bulimic symptoms, night eating, and loss‐of‐control eating. One study reported that foods consumed during binge‐eating episodes were entirely ultra‐processed. Findings for restrictive disorders were fewer and less consistent.

**Conclusions:**

Current evidence suggests a possible relationship between ultra‐processed food intake and nonrestrictive eating disorder symptoms and presentations. However, the small number of studies, their cross‐sectional design, and methodological heterogeneity limit stronger inferences. Longitudinal and experimental studies are needed to clarify mechanisms and inform future interventions.

## Highlights

- Evidence from seven cross‐sectional studies suggests a potential association between ultra‐processed food intake and nonrestrictive eating disorders, including bulimia nervosa and binge eating disorder.
- Findings were more consistent for symptoms involving binge eating and loss‐of‐control eating than for restrictive presentations.
- The limited and heterogeneous evidence underscores the need for longitudinal and experimental studies to clarify mechanisms linking ultra‐processed food intake to eating disorder symptoms.

## 1 Introduction

Eating disorders (EDs) represent a significant public health issue, characterised by dysfunctional eating behaviours that negatively affect individuals' physical and psychological health. Binge eating disorder (BED), bulimia nervosa (BN), and anorexia nervosa (AN) are the primary EDs, with a multifactorial aetiology involving genetic, psychological, social, and environmental factors (American Psychiatric Association 2014).

Additionally, patients with EDs often present other associated mental health conditions, such as depression, anxiety, obsessive‐compulsive disorder, borderline personality disorder, or other mood disorders (Keski‐Rahkonen and Mustelin 2016). These comorbidities frequently coexist and can influence the course and treatment of EDs.

EDs pose a considerable challenge to public health, affecting millions of people worldwide. In the United States, it is estimated that 9% of the population, or 28.8 million Americans, will develop an ED in their lifetime (Deloitte Access Economics 2024). Globally, the prevalence of EDs increased from 3.5% to 7.8% between 2000 and 2018 (Galmiche et al. 2019), reflecting a concerning rise in these disorders over the past few decades. These figures highlight the urgent need for effective interventions to prevent, diagnose, and treat EDs, aiming to mitigate their impact on the physical and mental health of affected individuals.

Since EDs are related to changes in dietary patterns (American Psychiatric Association 2014), understanding these modifications is crucial. Furthermore, there are studies suggesting that these changes may be associated with the consumption of ultra‐processed foods (UPFs) (Ayton and Ibrahim 2020).

UPFs are industrially produced foods containing little or no unprocessed ingredients, along with numerous additives. They are characterised by high levels of fats, sugars, and sodium, and are often hyperpalatable and calorie dense. UPFs are impractical to prepare in a domestic kitchen due to their complex processing and ingredient composition (Monteiro et al. 2019).

In recent years, the consumption of UPFs has increased due to their convenience, as many of these foods are ready to eat (Baker et al. 2020). Additionally, their low cost and long shelf life contribute to their popularity. However, the literature establishes that UPFs are linked to various adverse health effects. A recent meta‐analysis identified associations with 32 health parameters, including mortality, cardiovascular diseases, diabetes, obesity, and cancer (Lane et al. 2024).

However, the potential relationship between UPF consumption and mental health, including EDs, remains poorly explored. Understanding this connection is important for clarifying how dietary patterns may contribute to the development and maintenance of EDs.

Given the novelty of this topic and the heterogeneity of the available evidence, a scoping review is the most suitable approach to provide an overview of existing studies and highlight areas requiring further investigation. Therefore, the aim of this study was to map and synthesise the available evidence on the relationship between UPF intake and EDs in adolescents and adults, emphasising current knowledge gaps and directions for future research.

## 2 Methods

### 2.1 Design and Protocol

This scoping review was conducted in accordance with the methodological guidance proposed by the Joanna Briggs Institute (JBI) for scoping reviews and reported following the PRISMA‐ScR checklist. The review was previously registered on the Open Science Framework (DOI 10.17605/OSF.IO/EBU8Y). The concepts of interest were UPF intake and EDs.

### 2.2 Eligibility Criteria

The search for articles included in this review was guided by the research question: “What is known from the existing literature about the relationship between UPF intake and EDs in adolescents and adults?” The PCC framework (Population, Concept, Context) was used to organise the research question, search strategy, and eligibility criteria (Table 1).

**TABLE 1:** PCC criteria for inclusion of studies.

Parameter	Criterion
Population	Adolescents and adults of both sexes.
Concept	Consumption of ultra‐processed foods (UPFs) and eating disorders (EDs)
Context	Any setting or geographical region

All original articles published in English, Portuguese, or Spanish were considered. Studies involving adults and adolescents of both sexes were included if they addressed the concepts of interest, namely UPF intake and EDs. Both observational and experimental quantitative studies were eligible.

Studies were excluded if they were not available in full text, were published as abstracts in conference proceedings, involved pregnant women, or were conducted with hospitalised patients. UPF intake was considered valid when assessed using the Nova food classification or when clearly described as foods that fit the definition of UPFs.

### 2.3 Search Strategy

The following keywords and their respective synonyms were selected from the Health Sciences Descriptors (DeCS): eating disorders, anorexia, anorexia nervosa, bulimia, bulimia nervosa, binge eating disorder, industrialised foods, ultra‐processed foods, and fast foods.

Searches were conducted in PubMed, PsycINFO, and Embase. No filters were applied, and for each database, a high‐sensitivity strategy was developed with the assistance of a librarian. This review considered all articles available up to March 5, 2026, the date on which the search was performed. The search strategy was designed to be broad and inclusive, in line with the objectives of a scoping review.

A detailed description of the search strings used in each database is provided in Supporting Information S1.

### 2.4 Study Selection

Rayyan software was used to manage and screen records during the study selection process (Ouzzani et al. 2016). Two reviewers independently screened titles and abstracts according to the pre‐established eligibility criteria. After this initial stage, full texts were reviewed. In cases of disagreement, consensus was sought, and when necessary, a third reviewer acted as an arbiter. The process was conducted in line with recommendations for scoping reviews.

### 2.5 Data Extraction and Synthesis Process

Relevant information from the included studies was charted in an Excel spreadsheet by two independent reviewers and subsequently verified with a third reviewer The data charted included study label (author and year), country, design, sample characteristics (number of participants, sex, age), use of the Nova food classification, UPF intake, ED assessment, and main findings. Study authors were contacted by email to find out if any necessary information was missing from the article or if the information was unclear.

### 2.6 Risk of Bias and Quality of Evidence

In line with recommendations for scoping reviews, formal risk of bias assessment was not required. However, to provide additional descriptive information about the methodological characteristics of the included studies, two reviewers independently applied the Joanna Briggs Institute tool, the JBI Critical Appraisal Checklist for Analytical Cross‐Sectional Studies (Joanna Briggs Institute 2017). In case of discrepancies, consensus was sought with a third reviewer. The appraisal results are presented descriptively and were not used to exclude studies.

The tool consists of 8 items that can be answered with four response options: yes, no, uncertain, or not applicable. A “yes” score of ≥ 5 times, 3–4 times, and 0–2 times is considered to indicate high, moderate, and low methodological quality, respectively.

## 3 Results

### 3.1 Study Selection and Characteristics

A total of 930 articles were identified in the initial search, of which 137 were excluded as duplicates. Of the remaining 793 records screened by title and abstract, 770 did not meet the eligibility criteria. 23 full‐text articles were assessed, and 16 did not meet the criteria. Ultimately, seven studies were included in this scoping review. The study flowchart is shown in Figure 1. A summary of excluded studies after full‐text review and the reasons for their exclusion is provided in Supporting Information S1.

**FIGURE 1:**
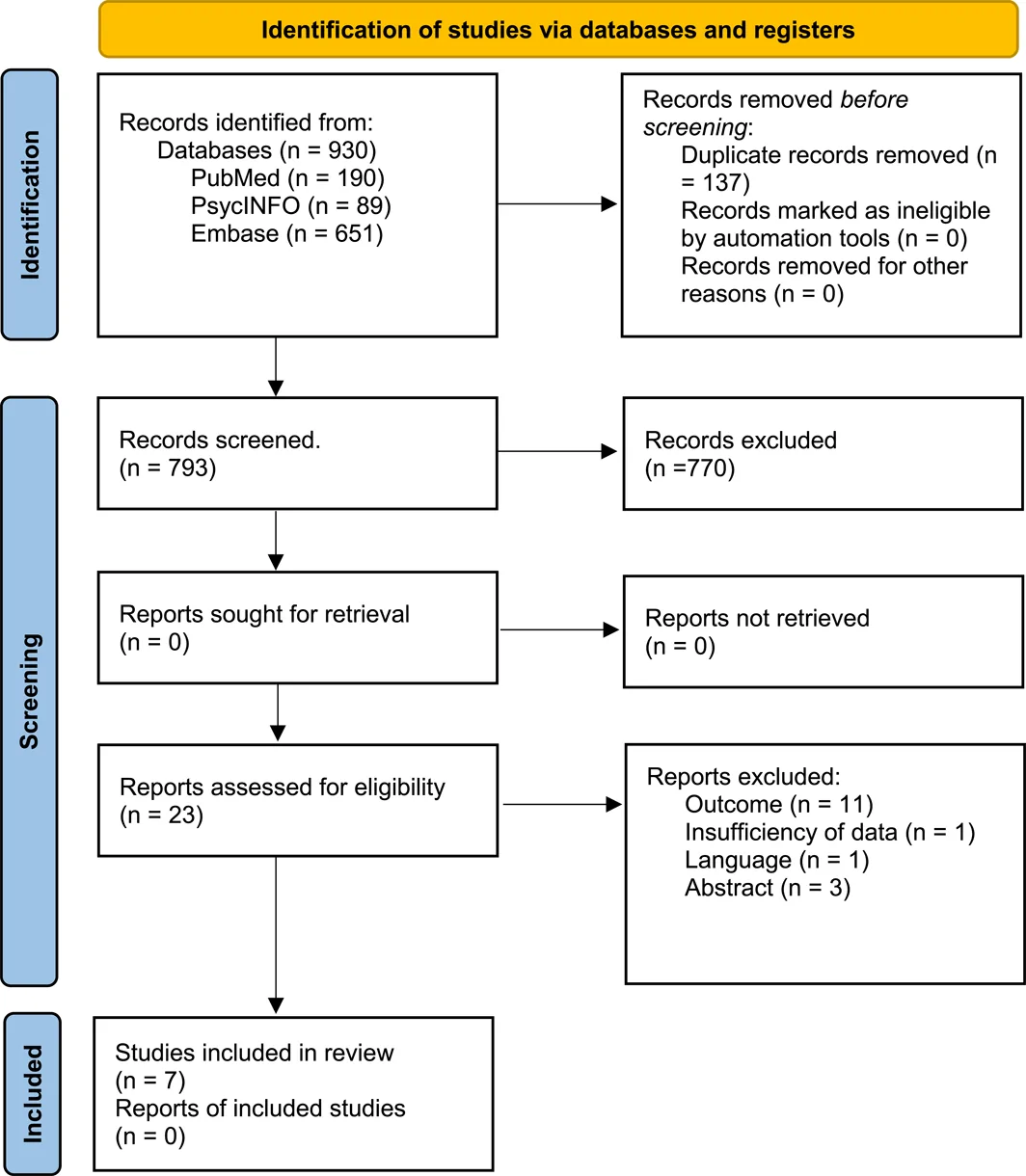
PRISMA flowchart of search and study selection. *Source:* Page MJ, et al. BMJ 2021; 372:n71. doi: 10.1136/bmj.n71. This work is licenced under CC BY 4.0. To view a copy of this licence, visit https://creativecommons.org/licenses/by/4.0/.

The seven included studies were all cross‐sectional and were conducted in the United Kingdom, France, Brazil, and Turkey. Together, they comprised 47,758 participants, including adolescents and adults. Most studies included adult samples, while one focused on adolescents with obesity. Sample sizes varied substantially, ranging from 73 to 43,993 participants. Most studies included predominantly female participants, although some studies enroled more balanced samples or samples with a higher proportion of men.

The included studies showed heterogeneity in the assessment of both UPF intake and EDs. Most studies used the Nova food classification directly or instruments derived from it to categorise UPFs, whereas one study assessed highly processed foods using a conceptually similar approach. EDs were identified using different methods, including DSM‐5 diagnoses, the SCOFF questionnaire combined with the Expali algorithm, and validated instruments assessing binge‐eating symptoms, bulimic symptoms, night eating syndrome, or broader ED symptomatology.

### 3.2 Outcomes

Across the included studies, associations between UPF intake and ED‐related outcomes were reported, although the outcomes and assessment methods varied across studies.

In the clinical sample from the United Kingdom, foods consumed during binge‐eating episodes were reported as entirely UPF. In the same study, individuals with anorexia nervosa, bulimia nervosa, and binge eating disorder reported diets in which approximately 55%, 72%, and 69% of foods consumed were classified as UPF, respectively (Ayton et al. 2021). In the French population‐based study, higher UPF intake was positively associated with bulimic disorders, binge eating disorders, and other ED, whereas no statistically significant association was observed for restrictive disorders (Figueiredo et al. 2022).

In a Brazilian adult sample, higher UPF consumption scores were observed among participants classified as having binge eating and other ED compared with those without ED symptoms. No significant differences were found for restrictive disorders or bulimia compared with the asymptomatic group (Walker et al. 2026). In adults with obesity, a higher proportion of calories derived from UPF was associated with higher scores for binge eating and bulimia symptoms, as well as with emotional, external, and uncontrolled eating, and with lower overall diet quality (Favaron et al. 2026). In the only included study involving adolescents, UPF intake was assessed in relation to binge eating symptoms, but no direct association between binge eating symptoms and UPF consumption was observed (Neres et al. 2025).

Among university students, higher intake of highly processed foods was associated with night eating syndrome, and correlations were also reported between night eating scores, digital addiction scores, and highly processed food consumption scores (Eroğlu et al. 2025). In adults seeking outpatient nutrition counselling, UPF intake was positively associated with ED symptomatology, and mediation analyses showed that ultra‐processed food addiction symptoms mediated 73.8% of the positive relationship between UPF intake and ED symptomology (Wiss and LaFata 2025).

A summary of these findings is presented in Table 2.

**TABLE 2:** Data extraction of the included studies.

Study label	Contry	Design	Aim	Sample (*N*/Sex/Age in years)	Nova food classification	UPF intake	ED dignosis	Results
Ayton_2021	United Kingdom	Cross‐sectional	Explore the degree of industrial processing in the food choices of patients with eating disorders.	73 participants (95,9% women). Participants were categorised as likely AN (*n* = 22 with mean age 24.7 ± 8.6); BN (*n* = 25 with a mean age 26.9 ± 7.1) and BED (*n* = 26 with a mean age 39 ± 10.8).	Yes	Self‐reported habitual intake over the past 2 weeks (including mealtimes, binging, and purging); Nova classification applied by two authors.	DSM‐5	Patients with AN consuming 55% UPF, as opposed to 72% in BN and 69% in BED patients, the difference was not statistically significant (*X* ^2^ test, *p* = 0.19). The foods patients consumed in a binge eating pattern were 100% UPF.
Figueiredo_2022	France	Cross‐sectional	Investigate whether UPF intake was associated with different ED types in a large population‐based study.	Total sample was 43.993 participants (76.1% women) of these 5.967 (13.6%) participants were categorised as likely ED being restrictive disorders (*n* = 444 with mean age 38.3 ± 13.5); bulimic disorders (*n* = 1.575 with mean age 43.3 ± 14.4); binge eating disorders (*n* = 3.124 with mean age 51.2 ± 13.6); other eating disorders (*n* = 824 with mean age 51.1 ± 14.9).	Yes	Mean intake from ≥ 2 24‐h dietary records.	SCOFF and Expali algorithm	The fully‐adjusted analyses revealed a positive association between UPF intake and bulimic disorders (OR = 1.08, 95% CI = 1.01–1.14, *p* = 0.02), binge eating (OR = 1.21, 95% CI = 1.16–1.26, *p* < 0.0001), and other ED (OR = 1.11, 95% CI = 1.02–1.20, *p* = 0.02). No significant association was detected for restrictive disorders (OR = 1.06, 95% CI = 0.95–1.18, *p* = 0.29).
Walker_2026	Brazil	Cross‐sectional	Examine the association between UPF consumption and ED symptoms in a large sample of Brazilian adults.	1760 participants with a mean age 29.99 ± 9.21 (53% men) of these 690 (39.2%) presented signs and symptoms of ED. Participants were categorised as likely ED being restrictive disorders (*n* = 26); bulimic disorders (*n* = 130); binge eating disorders (*n* = 453); other eating disorders (*n* = 81).	Yes	The Nova score of ultra‐processed food consumption for the Brazilian population was used (23 UPF subgroups); previous‐day self‐reported intake; score = sum of consumed subgroups (0–23).	SCOFF and Expali algorithm	The mean Nova score for UPF consumption was 3.15 (SD = 2.23); Nova scores for UPF consumption were significantly higher in participants with binge eating (*M* = 3.43, SD = 2.25) and other EDs (*M* = 3.85, SD = 2.58) compared with those without ED symptoms (*M* = 2.99, SD = 2.12; *p* < 0.05).
Favaron_2026	Brazil	Cross‐sectional	Assess the consumption of ultra‐processed foods and the presence of eating disorder symptoms in individuals with obesity.	77 volunteers with severe obesity, with a mean age of 36 ± 8.7 (77.9% women) of these 23.4% had high scores, indicating binge eating symptoms with possible presence of bulimia.	Yes	Mean dietary intake based on three 24‐h dietary recalls.	BITE	Volunteers were divided into groups of calories ingested from UPF (%): < 24.10% (*n* = 25); between 24.10% and 35.40% (*n* = 26); > 35.40% (*n* = 26). The consumption of UPF was positively associated with the symptoms of binge eating and bulimia (*p* = 0018, *β* = 0.008, 1.001 – 1.015 95% IC).
Neres_2025	Brazil	Cross‐sectional	Investigate associations between food intake (unprocessed/UPF), appetite mediators, and binge eating in adolescents with obesity.	96 adolescents with obesity (54 girls); 64.6% no binge eating, 35.4% with symptoms (7.3% severe, 28.1% moderate).	Yes	FFQ; frequency of consumption of 94 food items over the previous 6 months, self‐administered. Frequency data were converted into annual consumption scores. Participants were stratified into tertiles of UPF consumption score: T1 (0.48–4.25), T2 (4.29–6.68), and T3 (6.71–17.6).	BES	No significant association with binge eating symptoms (BES) and UPF consumption; BES median scores: *T*1 = 16 (1–46), *T*2 = 12 (3–32), *T*3 = 16.5 (0–30) (*p* = 0.376)
Eroğlu_2025	Turkey	Cross‐sectional	Investigate the relationship between NES, UPF consumption, and digital addiction in university students.	2512 university students with a mean age of 21.36 ± 2.04 (57.7% women) of these 20,9% (*n* = 524) presented the NES.	No	sQ‐HPF questionnaire (11 items; yes = 1 point; 0–11 total); ≥6 = high consumption;	NEQ	A positive correlation was detected between the NEQ scale total score and the UPF consumption scale total score (*r* = 0.095, *p* < 0.001).
Wiss_2025	USA	Cross‐sectional	To test mediation pathways between adverse childhood experiences and ED symptoms via post‐traumatic stress disorder symptoms, UPF intake frequency, and ultra‐processed food addiction.	304 participants with a mean age 39 ± 14.1 (74.3% women); of these 61.8% probable ED (positive screening).	Yes	FFQ; frequency of intake of seven UPF categories, ranging from never to ≥ 4 times/day. Total UPF frequency index: 0–56; dichotomised at the mean for descriptive analyses.	EDE‐QS	The mean UPF consumption score was 14.8 (SD = 8.5); higher UPF intake was positively associated with ED symptoms (*r* = 0.21, *p* < 0.01). UPF food addiction symptoms mediated 73.8% of the positive relationship between UPF intake and ED symptomology.

Abbreviations: AN, anorexia nervosa, BED, binge eating disorder, BES, Binge Eating Scale, BITE, Bulimic Investigatory Test Edinburgh, BN, bulimia nervosa, CI, confidence interval, DSM‐5, Diagnostic and Statistical Manual of Mental Disorders Fifth Edition, ED, eating disorder, EDE‐QS, Eating Disorder Examination Questionnaire Short Form, FFQ, Food Frequency Questionnaire, N, number of participants, NEQ, Night Eating Questionnaire, NES, night eating syndrome, OR, odds ratio, SCOFF, Sick‐Control‐One stone Fat‐Food, sQ‐HPF, Highly Processed Food Consumption Screening Questionnaire, T1, Tertile 1, T2, Tertile 2, T3, Tertile 3, UPF, ultra‐processed foods, USA, United States of America.

### 3.3 Risk of Bias and Quality of Evidence

Concerning methodological appraisal, all seven studies included in this review were classified as having high methodological quality according to the JBI Critical Appraisal Checklist for Analytical Cross‐Sectional Studies (Joanna Briggs Institute 2017). Scores ranged from 5 to 8 positive responses across the eight checklist items, indicating overall good methodological rigour despite some variation between studies. The appraisal results are presented in Table 3.

**TABLE 3:** Methodological quality of the studies included using the JBI Critical Appraisal Checklist for Analytical Cross‐Sectional Studies.

Study label	Item 1	Item 2	Item 3	Item 4	Item 5	Item 6	Item 7	Item 8	Quality rate	Methodological quality
Ayton_2021	No	Yes	Yes	Yes	No	No	Yes	Yes	5	High
Figueiredo_2022	Yes	Yes	Yes	Yes	Yes	Yes	Yes	Yes	8	High
Walker_2026	Yes	Yes	Yes	Yes	Yes	Yes	Yes	Yes	8	High
Favaron_2026	Yes	Yes	Yes	Yes	No	No	Yes	Yes	6	High
Neres_2025	Yes	Yes	Yes	Yes	Yes	Yes	Yes	Yes	8	High
Eroglu_2025	Yes	Yes	Yes	Yes	Yes	Yes	Yes	Yes	8	High
Wiss_2025	Yes	Yes	No	Yes	Yes	Yes	Yes	Yes	7	High

*Note:* Item 1: Were the criteria for inclusion in the sample clearly defined?; Item 2: Were the study subjects and the setting described in detail?; Item 3: Was the exposure measured in a valid and reliable way?; Item 4: Were objective, standard criteria used for measurement of the condition?; Item 5: Were confounding factors identified?; Item 6: Were strategies to deal with confounding factors stated?; Item 7: Were the outcomes measured in a valid and reliable way?; Item 8: Was appropriate statistical analysis used?.

## 4 Discussion

To our knowledge, this is the first scoping review to map the available evidence on the relationship between ultra‐processed food intake and eating disorders. In this review, seven cross‐sectional studies were included, comprising 47,758 participants from different countries and populations. However, the evidence was concentrated predominantly in adult samples, with only one included study involving adolescents. Although the evidence base remains limited, the available findings suggest a possible relationship between higher UPF intake and EDs symptoms and presentations, particularly those involving binge eating, bulimic symptoms, night eating, and loss‐of‐control eating. By contrast, findings for restrictive disorders were fewer and less consistent. At the same time, the exclusive reliance on cross‐sectional designs does not allow conclusions about temporality or causality, underscoring the need for longitudinal and experimental studies to clarify how this relationship may operate over time.

The included studies varied substantially in sample characteristics, methods used to assess UPF intake, and approaches to identifying EDs, yet some converging patterns emerged. In the clinical study by Ayton, individuals with anorexia nervosa, bulimia nervosa, and binge eating disorder reported diets in which approximately 55%, 72%, and 69% of foods consumed were classified as UPF, respectively, and foods consumed during binge‐eating episodes were entirely UPF (Ayton et al. 2021). In the large French study by Figueiredo, higher UPF intake was positively associated with bulimic disorders, binge eating disorders, and other eating disorders, whereas no significant association was found for restrictive disorders (Figueiredo et al. 2022). These findings are in line with a possible association between UPF intake and ED presentations involving binge eating.

Additional studies reported findings that were broadly compatible with this pattern, although not uniformly. In a Brazilian adult sample, higher UPF consumption was observed among individuals classified as having binge eating and other EDs, whereas no significant differences were found for restrictive disorders or bulimia compared with those without ED symptoms (Walker et al. 2026). In adults with obesity, higher UPF intake was associated with greater binge eating and bulimia symptoms, as well as higher emotional, external, and uncontrolled eating and lower diet quality (Favaron et al. 2026). By contrast, the only included study involving adolescents did not identify a direct association between UPF intake and binge eating symptoms (Neres et al. 2025). This finding is noteworthy because it differs from the pattern observed in several adult samples and suggests that the relationship between UPF intake and ED‐related outcomes may not be uniform across age groups. At the same time, this study was conducted in adolescents with obesity and focused specifically on binge eating symptoms, which makes direct comparison with the adult literature more difficult. Given that adolescence is a critical developmental period for the onset of EDs, the fact that only one included study examined this age group highlights an important gap in the current evidence base.

The included evidence also suggests that the possible relationship between UPF intake and ED‐related phenomena may extend beyond classical diagnostic categories such as AN, BN, and BED. Among university students, higher intake of highly processed foods was associated with night eating syndrome (Eroğlu et al. 2025), indicating that UPF exposure may also be relevant to disturbances involving recurrent evening or nocturnal eating. The study by Wiss and LaFata adds an important layer of complexity to the evidence base. In that sample of adults seeking outpatient nutrition counselling, UPF intake was positively associated with ED symptomatology, and symptoms of ultra‐processed food addiction mediated 73.8% of this positive association (Wiss and LaFata 2025). In addition, the analytical model incorporated adverse childhood experiences and symptoms of post‐traumatic stress disorder, indicating that the relationship between UPF intake and ED symptoms may, at least in some individuals, be embedded within a broader trauma‐related clinical context. Although these findings come from a specific outpatient sample and should be interpreted accordingly, they suggest that the role of UPF intake in ED symptomatology may involve not only eating behaviour itself, but also addictive‐like eating processes and co‐occurring psychological vulnerability.

Although the adverse associations between UPF consumption and physical health are well established in the literature (Pagliai et al. 2020), evidence regarding mental health remains more limited. A meta‐analysis published in 2022 examined the association between UPF consumption and mental health outcomes and reported associations with conditions such as anxiety and depression (Lane et al. 2022). Although that review did not specifically address EDs, it suggests that UPF consumption may also be relevant in the broader mental health context. At the same time, these relationships are likely to be complex and potentially bidirectional, since mental health symptoms may also influence food choice and reliance on readily available, highly palatable foods. In addition, individuals with EDs often present with co‐occurring mental health conditions (Keski‐Rahkonen and Mustelin 2016). It has been proposed that characteristics of UPFs, such as low fibre content, high energy density, and high levels of refined carbohydrates, among other features, may contribute to worse outcomes in conditions such as anxiety and depression (Marx et al. 2021). Whether similar mechanisms may be involved in EDs remains unclear.

The simultaneous increase in obesity and EDs has rarely been examined in an integrated way (Ayton and Ibrahim 2020). Although few studies have directly investigated the relationship between UPFs and EDs, the growing availability and consumption of these foods since the 1970s has occurred alongside increases in obesity and in disorders such as BN and BED (Ayton and Ibrahim 2020). In this context, it is also relevant to note that the association between UPF consumption and obesity is well established in the literature (Louzada et al. 2015). However, the relationship between obesity and EDs is also likely to be complex and potentially bidirectional, rather than reflecting a single causal pathway. ED symptoms may precede obesity, emerge in response to obesity, or interact with factors such as weight cycling and other psychosocial and metabolic influences. Individuals with obesity are reported to be 2.45 times more likely to develop disordered eating behaviours than those with normal weight (Ramaswamy and Ramaswamy 2023). In addition, an Australian population‐based study covering the period from 1995 to 2015 reported substantial increases in the prevalence of both obesity, from 19% to 33%, and binge eating, from 3% to 11%, with the largest increase observed for obesity with concomitant binge eating, which rose 7.3‐fold (Da Luz et al. 2017).

Furthermore, the exact mechanisms by which UPFs may contribute to EDs are not yet fully understood but may involve psychosocial, neurobiological, and environmental factors. Additionally, UPF consumption has significant metabolic consequences, including triggering insulin and glucose responses, stimulating appetite, and impacting various endocrine and neurobiological pathways, as well as the microbiome (Ayton and Ibrahim 2020). Moreover, the characteristics of UPFs, such as the rapid absorption of ingested calories, may contribute to excessive consumption of these foods (Forde et al. 2020).

Ultra‐processed foods have been suggested to influence brain systems involved in motivation and reward. They may also affect neural networks related to eating behavior, including pathways involving serotonergic and dopaminergic neurotransmission, as well as broader aspects of brain integrity and function (Contreras‐Rodriguez et al. 2022). Together with other psychosocial, metabolic, and environmental factors, these neurobiological alterations may be relevant to the observed association between UPF intake and EDs.

Among the limitations of the present review, it should be noted that all included studies used cross‐sectional designs, which does not allow causal or temporal inferences to be drawn from the findings. In addition, there was substantial heterogeneity in how both UPF intake and EDs were assessed across studies. While some studies relied on DSM‐5 diagnoses or Nova‐based dietary classification, others used screening instruments, symptom‐based measures, or tools conceptually aligned with UPF exposure rather than identical operational definitions. Although the included studies generally used recognized methods to reduce bias in the assessment of food consumption, some degree of measurement error is still possible, given the inherent challenges of dietary assessment. Another limitation is that the available evidence is concentrated predominantly in adult populations, with only one included study involving adolescents. This limits the extent to which the findings can be interpreted in younger age groups, despite adolescence being a critical developmental period for the onset of EDs. Another limitation is the limited number of available studies on this topic, which remains relatively underexplored. Finally, one study contributed the vast majority of the total sample, which may have disproportionately influenced the overall picture of the evidence. This imbalance highlights the need for additional studies with diverse populations, settings, and methodological approaches.

Despite these limitations, the present review contributes to the emerging literature on the possible relationship between UPF intake and EDs by synthesizing evidence from different populations and methodological approaches. Although stronger conclusions cannot yet be drawn, the available findings support the relevance of continuing to investigate UPFs within the broader context of eating‐disorder risk, symptoms, and maintenance. Given the global increase in UPF consumption and its established implications for physical and mental health, further research on this topic may be important for informing future prevention strategies, nutritional guidance, and clinical interventions.

## 5 Conclusion

This scoping review mapped the available evidence on the relationship between ultra‐processed food (UPF) consumption and eating disorders (EDs). The current literature suggests a possible association, particularly for nonrestrictive symptoms and presentations involving binge eating, bulimic symptoms, and loss‐of‐control eating, whereas evidence regarding restrictive disorders remains limited and less consistent. However, the small number of available studies, their cross‐sectional design, and the heterogeneity in methods used to assess both ultra‐processed food intake and eating disorders preclude stronger inferences. These findings highlight the need for additional longitudinal and experimental research to clarify temporal relationships, potential mechanisms, and the possible relevance of ultra‐processed food intake in the context of eating disorders.

## 6 Public Significance Statement

Ultra‐processed foods are widely consumed worldwide and are known to be associated with adverse physical health outcomes. This review maps the available evidence on their possible relationship with eating disorders, particularly nonrestrictive symptoms and presentations such as bulimia and binge eating. A better understanding of this relationship may help inform public health strategies aimed at promoting healthier food environments and guiding future research on dietary factors relevant to eating disorders.

## Author Contributions

**Marthina Streda Walker:** conceptualization, methodology, investigation, data curation, formal analysis, writing – original draft. **Natália R. Figueiredo:** methodology, investigation, data curation, formal analysis, writing – review and editing. **Luana Cristina Lermen:** investigation, data curation, writing – review and editing. **Maria Laura da Costa Louzada:** supervision, writing – review and editing. **Ana Maria P. Feoli:** conceptualization, methodology, supervision, writing – review and editing.

## Funding

This study was funded by the Coordenação de Aperfeiçoamento de Pessoal de Nível Superior (CAPES), Brazil, through a doctoral scholarship.

## Conflicts of Interest

The authors declare no conflicts of interest.

## Permission to Reproduce Material

No copyrighted material from other sources was reproduced in this article. All cited materials are properly referenced and used in accordance with copyright and fair use standards.

## Supporting information


Supporting Information S1
