# Supplementary material for: Ultra‐Processed Food Intake and its Association With Eating Disorders: A Scoping Review
Source: Eur Eat Disord Rev. 2026 Jul 4;34(6):1587–96. doi: 10.1002/erv.70144 (PMC13656770; doi:10.1002/erv.70144)
Supplement: Supplementary file 1 [file ERV-34-1587-s001.docx]

**Supplementary Material (S1)
Search strategy for each database:**

*Pubmed:* (“Industrialized Foods” OR “Ultra-Processed Foods” OR “Ultra Processed Foods” OR “Food, Ultra-Processed” OR “Food, Ultra Processed” OR “Foods, Ultra-Processed” OR “Ultra-Processed Food” OR “fast foods”[Mesh] OR “Fast Food” OR “Food, Fast” OR “Foods, Fast” OR “Convenience Foods” OR “Convenience Food” OR “Food, Convenience” OR “Foods, Convenience” OR “Ready-Prepared Foods” OR “Food, Ready-Prepared” OR “Foods, Ready-Prepared” OR “Ready Prepared Foods” OR “Ready-Prepared Food” OR “Ready-To-Eat Meals” OR “Ready To Eat Meals” OR “Meals, Ready-To-Eat” OR “Meal, Ready-To-Eat” OR “Meals, Ready To Eat” OR “Ready-To-Eat Meal”) AND (“Feeding and Eating Disorders”[Mesh] OR “Eating and Feeding Disorders” OR “Feeding Disorders” OR “Disorder, Feeding” OR “Disorders, Feeding” OR “Feeding Disorder” OR “Eating Disorders” OR “Disorder, Eating” OR “Disorders, Eating” OR “Eating Disorder” OR “Appetite Disorders” OR “Appetite Disorder” OR “Anorexia”[Mesh] OR Anorexias OR “Anorexia Nervosa”[Mesh] OR “Anorexia Nervosas” OR “Nervosa, Anorexia” OR “Nervosas, Anorexia” OR “Bulimia”[Mesh] OR Bulimias OR “Binge Eating” OR “Eating, Binge” OR “Bulimia Nervosa”[Mesh] OR “Nervosa, Bulimia” OR “Binge-Eating Disorder”[Mesh] OR “Binge Eating Disorder” OR “Binge-Eating Disorders” OR “Disorder, Binge-Eating” OR “Disorders, Binge-Eating” OR “Avoidant Restrictive Food Intake Disorder”[Mesh] OR ARFID)

*Psycinfo:* (“Industrialized Foods” OR “Ultra-Processed Foods” OR “Ultra Processed Foods” OR “Food, Ultra-Processed” OR “Food, Ultra Processed” OR “Foods, Ultra-Processed” OR “Ultra-Processed Food” OR “fast foods” OR “Fast Food”/exp OR “Food, Fast” OR “Foods, Fast” OR “Convenience Foods” OR “Convenience Food” OR “Food, Convenience” OR “Foods, Convenience” OR “Ready-Prepared Foods” OR “Food, Ready-Prepared” OR “Foods, Ready-Prepared” OR “Ready Prepared Foods” OR “Ready-Prepared Food” OR “Ready-To-Eat Meals” OR “Ready To Eat Meals” OR “Meals, Ready-To-Eat” OR “Meal, Ready-To-Eat” OR “Meals, Ready To Eat” OR “Ready-To-Eat Meal”) AND (“Feeding and Eating Disorders” OR “Eating and Feeding Disorders” OR “Feeding Disorders” OR “Disorder, Feeding” OR “Disorders, Feeding” OR “Feeding Disorder” OR “Eating Disorders” OR “Disorder, Eating” OR “Disorders, Eating” OR “Eating Disorder” OR “Appetite Disorders” OR “Appetite Disorder” OR “Anorexia” OR Anorexias OR “Anorexia Nervosa” OR “Anorexia Nervosas” OR “Nervosa, Anorexia” OR “Nervosas, Anorexia” OR “Bulimia” OR Bulimias OR “Binge Eating” OR “Eating, Binge” OR “Bulimia Nervosa” OR “Nervosa, Bulimia” OR “Binge-Eating Disorder” OR “Binge Eating Disorder” OR “Binge-Eating Disorders” OR “Disorder, Binge-Eating” OR “Disorders, Binge-Eating” OR “Avoidant Restrictive Food Intake Disorder” OR ARFID)

*Embase:* (“Industrialized Foods” OR “Ultra-Processed Foods” OR “Ultra Processed Foods” OR “Food, Ultra-Processed” OR “Food, Ultra Processed” OR “Foods, Ultra-Processed” OR “Ultra-Processed Food” OR “fast foods” OR “Fast Food”/exp OR “Food, Fast” OR “Foods, Fast” OR “Convenience Foods” OR “Convenience Food” OR “Food, Convenience” OR “Foods, Convenience” OR “Ready-Prepared Foods” OR “Food, Ready-Prepared” OR “Foods, Ready-Prepared” OR “Ready Prepared Foods” OR “Ready-Prepared Food” OR “Ready-To-Eat Meals” OR “Ready To Eat Meals” OR “Meals, Ready-To-Eat” OR “Meal, Ready-To-Eat” OR “Meals, Ready To Eat” OR “Ready-To-Eat Meal”) AND (“Feeding and Eating Disorders”/exp OR “Eating and Feeding Disorders” OR “Feeding Disorders” OR “Disorder, Feeding” OR “Disorders, Feeding” OR “Feeding Disorder” OR “Eating Disorders” OR “Disorder, Eating” OR “Disorders, Eating” OR “Eating Disorder” OR “Appetite Disorders” OR “Appetite Disorder” OR “Anorexia”/exp OR Anorexias OR “Anorexia Nervosa”/exp OR “Anorexia Nervosas” OR “Nervosa, Anorexia” OR “Nervosas, Anorexia” OR “Bulimia”/exp OR Bulimias OR “Binge Eating” OR “Eating, Binge” OR “Bulimia Nervosa”/exp OR “Nervosa, Bulimia” OR “Binge-Eating Disorder”/exp OR “Binge Eating Disorder” OR “Binge-Eating Disorders” OR “Disorder, Binge-Eating” OR “Disorders, Binge-Eating” OR “Avoidant Restrictive Food Intake Disorder”/exp OR ARFID)

**Supplementary Material (S2)**

**Table 1.** Summary of excluded studies after full-text review and reasons for non-inclusion.

| **Article** | **Reason for exclusion** |
| --- | --- |
| Ledoux et al., 2014 | Did not address UPF consumption in relation to EDs as defined by diagnostic criteria; focus was on fast-food availability and binge eating symptoms. |
| Harshman et al., 2019 | Did not address UPF consumption in relation to EDs; focused on dietary intake patterns in youth already diagnosed with ARFID. |
| Scaife et al., 2016 | Did not evaluate dietary intake or UPFs consumption; focused on neural responses to food images in anorexia nervosa. |
| Rasouli et al., 2021 | Focused on fast food intake as an environmental factor; did not evaluate UPFs consumption or its association with EDs specifically. |
| Ames et al., 2014 | Insufficient data on UPFs consumption; study focused on cognitive mechanisms and general snack/beverage intake. |
| Harvey et al., 2011 | Did not assess UPFs consumption; focused on frequency of meals and snacks in individuals with binge eating disorder. |
| Boggiano et al., 2014 | Assessed motives for eating palatable foods, not actual food consumption or dietary intake. |
| Pogodina et al., 2024 | Article published in a language other than English, Portuguese, or Spanish (Russian). |
| Hill et al., 2024 | Conference proceedings abstract (poster abstract published in conference abstracts/proceedings). |
| Favaron et al., 2024 | Conference proceedings abstract (poster abstract published in conference abstracts/proceedings). |
| Di Lorenzo et al., 2024 | Conference proceedings abstract (poster abstract published in conference abstracts/proceedings). |
| Luo et al., 2025 | Did not assess UPF consumption; evaluated broad food categories instead. |
| Barros et al., 2025 | Did not examine UPF consumption in relation to EDs; focus was eating rate. |
| Wiss et al., 2024 | Did not examine UPF consumption in relation to EDs; focus was weight suppression. |
| Sandri et al., 2024 | Did not assess UPF consumption according to NOVA or a clearly compatible definition; focused on dietary habits and ED symptoms in general. |
| Smith et al., 2025 | Did not assess UPF consumption; focused on highly processed foods (HPF) in relation to binge eating and food addiction symptoms. |

**Note:** UPF = Ultra-processed foods; Eds = eating disorders; ARFID = Avoidant/Restrictive Food Intake Disorder.

**References:**

Ames, S. L., Kisbu-Sakarya, Y., Reynolds, K. D., Boyle, S., Cappelli, C., Cox, M. G., Dust, M., Grenard, J. L., Mackinnon, D. P., & Stacy, A. W. (2014). Inhibitory control effects in adolescent binge eating and consumption of sugar-sweetened beverages and snacks. *Appetite, 82*, 180–192. <https://doi.org/10.1016/j.appet.2014.06.013>

Barros, L. de M., Peixoto, V. A., Carvalho, G. C. O. de, Pereira, M. R., Carnaúba, R. T. L., & Bueno, N. B. (2025). Association between psychobehavioral factors and the increased eating rate of ultra-processed versus non-ultra-processed meals in individuals with obesity: A secondary analysis of a randomized trial. *Nutrients, 17*, 2236. <https://doi.org/10.3390/nu17132236>

Boggiano, M. M., Burgess, E. E., Turan, B., Soleymani, T., Daniel, S., Vinson, L. D., Lokken, K. L., Wingo, B. C., & Morse, A. (2014). Motives for eating tasty foods associated with binge-eating: Results from a student and a weight-loss seeking population. *Appetite, 83*, 160–166. <https://doi.org/10.1016/j.appet.2014.08.026>

Di Lorenzo, M., Gautiero, C., Cacciapuoti, N., Lonardo, M., Pistone, L., Micanti, F., & Guida, B. (2024). *Ultraprocessed food intake and eating behaviour in bariatric surgery candidates* (GC2.122) [Conference abstract]. *Obesity Facts, 17*(Suppl. 1), 212. <https://doi.org/10.1159/000538577>

Favaron, C. M., Mônico-Neto, M., Galvão, T. D., Antunes, H. K. M., Bittencourt, L. R. A., Tufik, S., & Campos, R. M. S. (2024). *Impact of ultra-processed foods consumption on the presence of eating disorders symptoms and unusual eating behavior in adults with obesity* (P188) [Conference abstract]. *Obesity Reviews* (Suppl. 1). https://doi.org/10.1111/obr.13788

Harshman, S. G., Wons, O., Rogers, M. S., Izquierdo, A. M., Holmes, T. M., Pulumo, R. L., Asanza, E., Eddy, K. T., Misra, M., Micali, N., Lawson, E. A., & Thomas, J. J. (2019). A diet high in processed foods, total carbohydrates and added sugars, and low in vegetables and protein is characteristic of youth with avoidant/restrictive food intake disorder. *Nutrients, 11*(9), 2013. <https://doi.org/10.3390/nu11092013>

Harvey, K., Rosselli, F., Wilson, G. T., DeBar, L. L., & Striegel-Moore, R. H. (2011). Eating patterns in patients with spectrum binge eating disorder. *International Journal of Eating Disorders, 44*(5), 447–451. <https://doi.org/10.1002/eat.20839>

Hill, E., Khandpur, N., Siebert, J., Ostendorf, D., Zhaoxing, P., Scorsone, J., Wayland, L., Bing, K., Breit, M., MacLean, P., Melanson, E., Bessesen, D., Borengasser, S., & Catenacci, V. (2024). *Ultra-Processed Food Intake is Associated with Eating Behaviors Prior to and During Weight Loss* (Poster-011) [Conference abstract]. *Obesity* (Suppl. 1), 59. https://doi.org/10.1002/oby.24195

Ledoux, T. A., Adamus-Leach, H., O’Connor, D. P., Mama, S. K., & Lee, R. E. (2014). The association of binge eating and neighbourhood fast-food restaurant availability on diet and weight status. *Public Health Nutrition, 18*(2), 352–360. <https://doi.org/10.1017/S1368980013003546>

Luo, Y., Morales, J. C., Dunton, G. F., & Mason, T. B. (2025). Assessment of EMA binge-eating symptoms in adolescents: Factor analysis and associations with social context and food types. *Appetite, 214*, 108212. <https://doi.org/10.1016/j.appet.2025.108212>

Pogodina, A. V., Astakhova, T. A., Lebedeva, L. N., & Rychkova, L. V. (2024). *Eating patterns and risk of eating disorders in adolescents*. *Voprosy pitaniia [Problems of Nutrition]*, 93(3), 31–40. <https://doi.org/10.33029/0042-8833-2024-93-3-31-40>

Rasouli, A., Mohiti, S., Javadi, M., Panjeshahin, A., Kazemi, M., & Shiri-Shahsavar, M. R. (2021). The effect of daily fast food consumption, family size, weight-caused stress, and sleep quality on eating disorder risk in teenagers. *Sleep and Breathing, 25*(3), 1527–1533. <https://doi.org/10.1007/s11325-020-02189-9>

Sandri, E., Sguanci, M., Cantín Larumbe, E., Cerdá Olmedo, G., Piredda, M., & Mancin, S. (2024). Influence of nutrition, lifestyle habits, and socio-demographic determinants on eating disorder symptoms in the Spanish young adult population: A cross-sectional nationwide survey. Medicina, 60(10), 1565. https://doi.org/10.3390/medicina60101565

Scaife, J. C., Godier, L. R., Reinecke, A., Harmer, C. J., & Park, R. J. (2016). Differential activation of the frontal pole to high vs low calorie foods: The neural basis of food preference in Anorexia Nervosa? *Psychiatry Research: Neuroimaging, 258*, 44–53. <https://doi.org/10.1016/j.pscychresns.2016.10.004>

Smith, K. E., Khalil, Z., Li, S., & Mason, T. B. (2025). Exploring reward, weight, and processed foods as mechanisms of binge eating and food addiction. Eating Behaviors, 58, 102019. https://doi.org/10.1016/j.eatbeh.2025.102019

Wiss, D. A., LaFata, E. M., & Tomiyama, A. J. (2024). A novel weight suppression score associates with distinct eating disorder and ultra-processed food addiction symptoms compared to the traditional weight suppression measure among adults seeking outpatient nutrition counseling. *Journal of Eating Disorders, 12*, 75. <https://doi.org/10.1186/s40337-024-01029-5>
